# EDITORIAL COMMENT: ROBOTIC URETEROURETEROSTOMY FOR TREATMENT OF A PROXIMAL URETERIC STRICTURE

**DOI:** 10.1590/S1677-5538.IBJU.2015.0249.1

**Published:** 2016

**Authors:** Lucas Wiegand

**Affiliations:** 1 University of South Florida Morsani College of Medicine, Tampa, FL, USA

Ureteral reconstruction can be challenging when done open or in a minimally-invasive manner. Ureteral reconstruction is a perfect use of the surgical robot. Here Andrade et al. (1) present a well-made and illustrative video that demonstrates the steps needed to complete a technically sound, minimally-invasive proximal ureteral repair. This will add to the body of literature and video instruction that improves and propels the field of robotic/minimally-invasive urologic reconstruction.
